# Metabolic reprogramming and immunosenescence: a new sight for glioma therapy

**DOI:** 10.3389/fcell.2026.1754980

**Published:** 2026-01-28

**Authors:** Huali Fan, Shizhuo Yang, Qing Lu, Liming Chang

**Affiliations:** 1 Department of Pharmacy, West China Tianfu Hospital, Sichuan University, Chengdu, China; 2 Department of Pharmacy, West China Hospital, Sichuan University, Chengdu, China

**Keywords:** epigenetic regulation, glioma, immunosenescence, metabolic reprogramming, tumor microenvironment

## Abstract

Gliomas, the most prevalent primary tumor of the central nervous system, are characterized by a poor prognosis and a high recurrence rate. The glioma microenvironment is highly immunosuppressive, which poses a major obstacle to effective immunotherapy. Metabolic reprogramming is a hallmark of glioma, driving tumor progression and therapy resistance. Key alterations include the Warburg effect, increased glutamine dependency, enhanced pentose phosphate pathway activity, and dysregulated lipid metabolism. Immunosenescence, the age-dependent decline in immune function that contributes to disease pathogenesis, encompasses immune dysregulation, senescence-associated secretory phenotype (SASP) accumulation, and epigenetic changes, which together drive immune cell dysfunction and foster an immunosuppressive microenvironment. Meantime, senescent immune cells may change the metabolic microenvironment, whereas metabolic reprogramming also influence immune system. Thus, this small essay is on the purpose of demonstrating the significance and function of metabolic reprogramming and immunosenescence in gliomas, providing evidence of promising therapeutic strategies.

## 1 Introduction

Gliomas, one of the most common primary brain tumors (for ∼30%), develop from neural stem or progenitor cells carrying tumor-initiating genetic alteration (Weller et al., 2024). According to the fifth edition of the WHO Classification of Tumors of the Central Nervous System (CNS) in 2021, gliomas are divided into five major groups: adult-type diffuse gliomas; pediatric-type diffuse low-grade gliomas; pediatric-type diffuse high-grade gliomas; circumscribed astrocytic gliomas; and ependymal tumors based on histological features and distinct molecular biomarkers (Louis et al., 2021). Equally, gliomas are classified into grades 1–4, and the malignant glioblastoma (GBM) belongs to grade 4 with a median overall survival time slightly over a year (Tao et al., 2023; Han et al., 2025). The mutations in the genes for isocitrate dehydrogenase type 1 (IDH1) or histone H3 are typical driver mutations in gliomas, which lead to weighty epigenetic changes, genomic instability, and subsequent acquisition of additional tumor-promoting genetic alterations, or deletions or truncating mutations of tumor suppressor genes (Bunse et al., 2025). Except for genetic factors and external risk factors (exposure to ionizing radiation), Advanced age is associated with gliomas, especially GBM (median age: 66 years) (Han et al., 2024; Weller et al., 2024). Currently, the therapeutic strategies for gliomas are surgery combined with chemotherapy or radiotherapy, and exploring innovative techniques such as immunotherapy and the integration of medical and engineering technology therapy, as well as natural compounds (Lin et al., 2024; Han et al., 2025; Wang et al., 2025). Traditionally, the CNS is an immunologically privileged organ as a result of the presence of the blood-brain barrier (BBB) and the absence of lymphatic vessels. In glioma progression, the BBB becomes impaired, thereby enabling immune cells to transmigrate across the BBB, whereas the interaction between immunocytes and glioma cells led to an immunosuppressive tumor microenvironment (TME), resulting in poor response to immunotherapy (Pu et al., 2025). Additionally, due to the deficient prognosis and high recurrence rate, investigation for promising strategies is significant.

**FIGURE 1:**
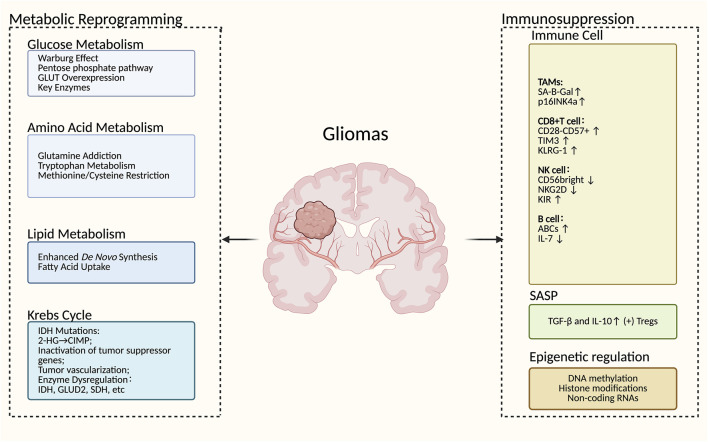
The overview of metabolic reprogramming and immunosenescence in gliomas. The metabolic reprogramming of gliomas mainly includes glucose metabolism, amino acid metabolism, lipid metabolism, and the Krebs cycle. Immunosenescence primarily involves alterations in immune cells, secretion of SASP, and epigenetic modifications. Supported by BioRender (https://app.biorender.com).

Metabolic reprogramming represents a hallmark of gliomas, facilitating rapid cancer cell proliferation and immune evasion through remodeling of the tumor microenvironment (Figure 1). A classic manifestation of this reprogramming is the Warburg effect, characterized by cancer cells exhibiting extremely high glycolytic rate and lactate production capacity even under aerobic conditions (Ekici et al., 2022). Beyond aerobic glycolysis, the glutamine uptake rate of gliomas is significantly increased, and compared with healthy cells, they require more glutamine storage to maintain survival because glutamine serves as a crucial energy substrate and carbon source for cancer cells (Pavlova and Thompson, 2016). Proliferating cells and tumor cells must also shunt carbon from glycolysis into the pentose phosphate pathway (PPP) for nucleotide synthesis and combating oxidative stress through upregulating PPP-related enzymes and downregulating glycolytic enzymes (Langbein et al., 2006). To meet the high energy demand, tumor cells enhance their ability to take up lipids from the environment and increase lipid biosynthesis (Cortes Ballen et al., 2024). GBM cells also utilize mitochondrial glucose oxidation to promote active growth *in vivo* (Agnihotri and Zadeh, 2016). In gliomas, metabolic reprogramming fosters multidrug resistance *via* multidimensional mechanisms: adaptive alterations in energy metabolism, redox homeostasis, stemness regulation, and microenvironment remodeling. For instance, aerobic glycolysis activation promotes HIF-1α signaling, reducing drug penetration and enhancing efflux; glutamine metabolism supports reactive oxygen species (ROS) clearance and epigenetic modification, while lipid metabolism bolsters stemness and immune microenvironment remodeling (Hsu, 2023; D’Aprile et al., 2025; Vijayanathan and Ho, 2025). In-depth analysis of the molecular mechanisms underlying metabolic reprogramming in gliomas and identification of potential therapeutic targets have important theoretical and clinical significance for developing novel therapeutic strategies and improving patient prognosis.

With advancing age, the immune system is abnormally activated or disrupted, resulting in remodeling and decline of immune function, named immunosenescence, which was proposed by Roy Walford in the 1960s (Fu et al., 2025). Immunosenescence is primarily driven by bone marrow aging and thymic involution; it can be further exacerbated by DNA damage related to various signaling pathways resulting from diverse endogenous and exogenous factors (Zhang et al., 2024). Immunosenescence is characterized by several key alterations, such as senescent immune cells, thymic involution, disrupted naïve/memory ratio in T and B cells, inflammaging, and metabolic dysregulation, among others (Liu Z. et al., 2023). Thymic involution underlies the imbalance in immune cell proportions, particularly for T cells. Meanwhile, the activity of other immune cells, such as macrophages and natural killer (NK) cells also reduces to accelerate the development of suppressive TME (Nguyen and Cho, 2025). The central pillar of aging is inflammaging, referred to as a systemic state of chronic low-grade inflammation. The senescence-associated secretory phenotype (SASP) of senescent cells, characterized by the secretion of numerous pro-inflammatory factors such as interleukin-1 (IL-1), IL-6, and tumor necrosis factor (TNF) promotes the inflammaging phenotype, and the components of SASP reprogram the TME, suppress CD8^+^ T cell activity, thereby fostering an immunosuppressive TME (Liu Z. et al., 2023; Fu et al., 2025). As a result, targeting immune aging might reverse the suppressive TME and improve the immunotherapy of gliomas.

Thus, this review aims to illustrate the features of metabolic reprogramming and immunosenescence in gliomas, with the ultimate goal of informing the development of potent glioma therapeutics.

## 2 Metabolic reprogramming of gliomas

Metabolic reprogramming refers to the process by which tumor cells, to meet the demands of rapid proliferation and adapt to the harsh microenvironment, reshape cellular metabolic pathways by regulating the activity of key metabolic enzymes and signaling pathways. Metabolic reprogramming events in gliomas are not only adaptive mechanisms of the tumor that help it continuously meet the growing demand for biosynthetic molecules and energy, but also promote the production of metabolites that are closely associated with multiple oncogenic signaling pathways (Trejo-Solis et al., 2023).

### 2.1 Warburg effect and PPP

#### 2.1.1 Warburg effect

A hallmark of cancer cell dysregulated metabolism is the “Warburg effect” or aerobic glycolysis, a phenomenon first described by Otto Warburg, in which cells preferentially metabolize glucose to lactate in the cytoplasm despite available oxygen (Callao and Montoya, 1961). This metabolic preference is attributed to the rapid ATP yield afforded by glycolysis and the diversion of glycolytic intermediates into biosynthetic pathways to generate biomass essential for proliferation. Furthermore, lactate production helps maintain cellular redox balance, permitting sustained glycolytic flux. Lactate acidifies the brain microenvironment and, through the action of matrix metalloproteinases (MMPs), degrades the extracellular matrix, thereby fostering tumor invasion (Venneti and Thompson, 2017).

##### 2.1.1.1 Key regulatory factors of Warburg effect

A spectrum of molecules serves as key regulators of glycolysis, including glucose transporter (GLUT), enzymes (HKs, PFK-1, PKs, G6PI, GA3PDH), and transcription factors (HIF-1α, c-Myc, p53). Glycolysis initiates with the uptake of glucose from the extracellular milieu into the cytosol, a process primarily mediated by glucose transporter (GLUT) family proteins. In GBM, the overexpression of GLUT-1 and GLUT-3 was a notable feature, and their elevated levels are associated with poorer patient survival. Notably, studies by Libby et al. demonstrated that GLUT-3 overexpression specifically promoted tumor invasiveness (Libby et al., 2021; Chamarthy and Mekala, 2023). Functionally, mutation of the GLUT-1 CYS207 residue to serine abrogated its palmitoylation and subsequent membrane localization, resulting in suppressing glycolysis, impeding cancer cell proliferation, and inhibiting the tumorigenic potential of GBM (Zhang Z. et al., 2021). The glycolytic process generated substantial lactate, which was predominantly extruded by monocarboxylate transporter 1 (MCT1), particularly in IDH-wildtype gliomas. When knockdown MCT1, the sensitivity of GBM cells to temozolomide (TMZ) markedly increased, and survival was extended (Miranda-Gonçalves et al., 2013; Miranda-Gonçalves et al., 2021). MCT1 was the key proton-coupled lactate exporter in gliomas, essential for sustaining intracellular pH and glycolysis. In IDH1-mutant gliomas, 2-hydroxyglutarate (2-HG) epigenetically suppressed MCT1 expression, thereby impairing lactate efflux (Choi et al., 2012; Fu et al., 2015).

Hexokinase (HK) is a key enzyme in the first step of glycolysis, phosphorylating glucose to glucose-6-phosphate (G-6-P). Hexokinase 2 (HK2) is the most well-characterized isoform in the HK family and serves as the major isoform in brain tumors, such as GBM (Wolf et al., 2011b; Di Magno et al., 2014). Notably, HK2 was preferentially expressed in GBM compared to low-grade astrocytomas and normal brain tissues, and its expression level correlated with patient prognosis (Agnihotri and Zadeh, 2016; Huang et al., 2022). Genetic depletion of HK2 attenuated aerobic glycolysis, restored oxidative phosphorylation, and promoted apoptosis, consequently improving survival in GBM xenograft models (Wolf et al., 2011a; Wolf et al., 2011b). Phosphofructokinase-1 (PFK-1) is involved in a key regulatory and rate-limiting step of glycolysis. 6-phosphofructo-2-kinase/fructose-2,6-bisphosphatase 3 (PFKFB3)is one of the isoforms of phosphofructokinase-2 (PFK-2). Inhibition of PFKFB3 expression or activity reduced the level of fructose-2,6-bisphosphate (F-2,6-BP) to suppress PFK-1 activity and glycolytic rate, and ultimately inhibited glioma cell proliferation (Yang et al., 2024). Furthermore, studies indicated that transforming growth factor-β1 (TGF-β1) upregulated the mRNA and protein level expression of PFKFB3 in glioma cells to enhance glycolytic flux, glucose uptake, and lactate production *via* activating p38 mitogen-activated protein kinase (MAPK) and phosphoinositide 3-kinase (PI3K)/protein kinase B (Akt) signaling pathway (Rodríguez-García et al., 2017).

Pyruvate kinase (PK) is a key enzyme in the payoff phase of glycolysis, mediating the conversion of phosphoenolpyruvate and ADP to pyruvate and adenosine triphosphate (Venneti and Thompson, 2017). In the developing brain, the predominant PKM2 variant facilitates anabolism through its low-activity state, while the glycolytic enzyme Glucose-6-phosphate isomerase (G6PI) exerts multifaceted functions, also acting as an autocrine motility factor (AMF) (Wolf et al., 2011b; Israelsen et al., 2013; Jeffery et al., 2000). Compared to GBM patients without AMF expression, the overall survival rate of GBM patients with AMF overexpression was lower (Tanizaki et al., 2006). Glyceraldehyde-3-phosphate dehydrogenase (GA3PDH) plays an important role in the Warburg effect, catalyzing the conversion of glyceraldehyde-3-phosphate (GA3P) to 1,3-bisphosphoglycerate (1,3BPG) in the glycolytic pathway, and GA3PDH was significantly upregulated in GBM biopsy specimens relative to low-grade gliomas and normal brain tissues (Appelskog et al., 2004; Akram, 2013). Lactate dehydrogenase (LDH) catalyzes the conversion of pyruvate to lactate under anaerobic conditions, while oxidizing reduced nicotinamide adenine dinucleotide (NADH) to its oxidized form (NAD^+^). In GBM, overexpression of the lactate dehydrogenase A (LDHA) isoform was frequently observed, channeling glycolytic flux toward lactate production and thereby promoting tumor cell survival and proliferation (Daniele et al., 2015).

Hypoxia-inducible factors (HIFs) are key regulators of the cellular response to hypoxia in GBM, among which hypoxia-inducible factor 1α (HIF-1α) plays a central role in regulating gene expression when oxygen levels decrease. Besides, HIF-1α is a critical transcription factor that controls the expression of genes related to glucose metabolism. In GBM cells, HIF-1α was stabilized under hypoxic conditions, which in turn led to increased expression of glycolytic enzymes and glucose transporters, thereby promoting the Warburg effect (Venneti and Thompson, 2017; Cortes Ballen et al., 2024). In GBM, glycolytic flux was enhanced through the dysregulation of key metabolic regulators, including p53 and signal transducer and activator of transcription 3 (STAT3). STAT3 activation upregulated the expression of transcription factors such as HIF-1α and c-Myc, which collectively promoted GBM cell proliferation (Yu et al., 2017; Papavassiliou and Papavassiliou, 2022).

#### 2.1.2 Pentose phosphate pathway (PPP)

Beyond enhanced glycolysis, proliferating and tumor cells redirect glycolytic carbon flux into the PPP to support nucleotide biosynthesis and counteract oxidative stress (Agnihotri and Zadeh, 2016). The PPP converts G-6-P into ribose-5-phosphate, which is an essential precursor for nucleotide assembly, and generates reducing equivalents in the form of nicotinamide adenine dinucleotide phosphate (NADPH). Subsequently, ribose-5-phosphate is utilized in purine synthesis through the incorporation of glutamine, glycine, aspartate, carbon dioxide, and tetrahydrofolate, or serves as the backbone for pyrimidine formation along with bicarbonate, aspartate, and glutamine. The phenotypic switch between migration and proliferation in tumor cells was critically regulated by the metabolic shift from the PPP to glycolysis. This shift was driven by synergistic signals from the tumor microenvironment, including mechanics and oxygen tension. On the one hand, elevated matrix stiffness activated integrin-focal adhesion kinase (FAK)-YAP/TAZ signaling, which downregulated key PPP enzymes, thereby diverting metabolic flux toward glycolysis and promoting the proliferative over the migratory phenotype (Romani et al., 2022). On the other hand, under hypoxic conditions, GBM cells suppressed the expression of key PPP enzymes, including glucose-6-phosphate dehydrogenase (G6PD), 6-phosphogluconate dehydrogenase (PGD), and transketolase (TKT), while upregulating glycolytic enzymes such as HK2, 6-phosphofructokinase platelet type (PFKP), aldolase C (ALDOC), PKM2, and LDHA. This metabolic rewiring promoted a phenotypic shift toward enhanced migration at the expense of reduced proliferation (Vijayanathan and Ho, 2025).

### 2.2 Metabolic reprogramming of amino acids

Amino acids (AAs) serve as fundamental building blocks for proteins and are essential regulators of cellular division, differentiation, and function. Dysregulation of amino acid metabolism is a common feature in numerous pathological conditions, including GBM and other tumors (Ling et al., 2023; Lu et al., 2024).

Glutamine (Gln), the most abundant non-essential amino acid in the human body, serves as a pivotal biosynthetic precursor and a critical source of NADPH and glutathione for maintaining redox homeostasis (Bergström et al., 1974; Curi et al., 2005; Lee et al., 2018). In the brain, the production of the neurotransmitters glutamate and γ-aminobutyric acid (GABA) depends on the neuron-astrocyte metabolic coupling known as the glutamine-glutamate cycle (Bak et al., 2006). Gliomas co-opted this essential pathway, exhibiting markedly enhanced glutamine uptake and dependency to sustain their survival and proliferation, with elevated glutamine concentrations correlating with tumor progression (Ekici et al., 2022; Venneti et al., 2015). This metabolic reprogramming was driven by key regulatory factors: altered glutamate transporter expression [upregulated Glutamate/Aspartate Transporter (GLAST) and downregulated Glutamate Transporter 1 (GLT1)] led to extracellular glutamate accumulation, potentially modulating the tumor microenvironment; oncogenic Myc protein upregulated glutamine transporters and anabolic enzymes; and the balance between glutamine-synthesizing (glutamine synthetase, GS) and glutamine-catabolizing enzymes (glutaminase, GLS) was disrupted, with the oncogenic isoform GLS1 promoting glutamine addiction (McCormick et al., 1990; de la Rosa et al., 2009; Matés et al., 2013; Pavlova and Thompson, 2016; Matés et al., 2019; Matés et al., 2020; Trejo-Solis et al., 2023). Furthermore, glutamine could activate mTOR complex 1 (mTORC1), a key component of the PI3K-Akt-mTOR signaling pathway, which was known to regulate critical cellular processes such as cell growth, proliferation, and survival of gliomas (Obara-Michlewska and Szeliga, 2020; Garcia et al., 2021).

The metabolic pathways of tryptophan (Trp) primarily encompass the kynurenine (Kyn) pathway, the serotonin pathway, and the indole pathway. Oldak et al. reported that levels of Trp metabolites in grade 4 gliomas were significantly higher than those in grade 2–3 gliomas (Oldak et al., 2024). Indoleamine 2,3-dioxygenase 1 (IDO1) was highly expressed in glioma stem cells, and its upregulation was associated with enhanced chemoresistance in GBM cells. Furthermore, the administration of IDO1 antagonists had been shown to improve TMZ cytotoxicity in a mouse glioma model (Hanihara et al., 2016; Ozawa et al., 2020). Overexpression of tryptophan 2,3-dioxygenase 2 (TDO2) in the Kyn pathway within glioma cells augmented Trp metabolism, thereby facilitating the release of biologically active metabolites such as 3-hydroxykynurenine, L-kynurenine, 3-hydroxybenzoic acid, and quinolinic acid. These metabolites promoted the proliferation and tumorigenic potential of glioma cells by activating the aryl hydrocarbon receptor (AhR)/Akt signaling pathway (Zhong et al., 2022). In contrast to the tumor-promoting effects of the kynurenine pathway, melatonin from the serotonin pathway exerted antitumor effects, while activation of the indole pathway enzyme interleukin-4-induced 1 (IL4i1) promoted cancer cell survival (Srivastava et al., 2025). R-2-hydroxyglutarate (R-2-HG), the neomorphic enzymatic product of mutant IDH, could significantly strengthen the activity of TDO2, indirectly activating the kynurenine pathway in monocytes/macrophages. Furthermore, in IDH-mutant gliomas, the extracellular Trp concentration is significantly higher than that in IDH-wildtype tumors and normal brain tissues. This elevated Trp spatially overlapped with the distribution of R-2-HG, forming a “tryptophan-enriched microenvironment” that sustained the activation of key metabolic pathways, ultimately influencing immune response (Friedrich et al., 2021).

GBM cells rewire their amino acid metabolism to fuel tumorigenesis. They primarily imported serine and glycine via the AA transporter alanine serine cysteine transporter 2 (ASCT2) (SLC1A5) to feed the serine-glycine-one-carbon (SGOC) pathway, which was sustained by the conversion of serine to glycine by serine hydroxymethyltransferase (SHMT) and the *de novo* serine synthesis from 3-phosphoglycerate (3-PG) by phosphoglycerate dehydrogenase (PHGDH) and phosphoserine phosphatase (PSPH). Notably, elevated PSPH and SHMT1 levels correlated with poor patient survival (Srivastava et al., 2025; Chen S. et al., 2022). Concurrently, arginine drove tumor growth, survival, and proliferation by serving as a precursor for nitric oxide, polyamines, and other metabolites, and further promoted invasion by enhancing cellular adhesion (Hou et al., 2022). The upregulation of the cysteine catabolic pathway bolstered antioxidant defense through glutathione synthesis (Noch et al., 2024). In contrast to these pro-tumorigenic pathways, methionine exerted a suppressive effect by inhibiting key signaling proteins like PI3K, thereby curbing GBM cell growth (Palanichamy et al., 2016; Palanichamy and Chakravarti, 2017). In glioma cells, the rewiring of amino acid metabolism (notably the increased uptake of glutamine, serine, and cysteine) underlay a heightened capacity for GSH synthesis (Li and Zhang, 2016; Yang and Stockwell, 2016); together with pathways like NRF2, this established a potent GSH-dependent antioxidant system that managed intrinsic oxidative stress to promote survival and growth while conferring resistance to radiotherapy, chemotherapy, and ferroptosis (Trachootham et al., 2009; Seibt et al., 2019; Fan et al., 2017).

### 2.3 Metabolic reprogramming of lipids

Beyond their role as energy reserves in the form of triglycerides (simple lipids), lipids are vital structural constituents of cell membranes, primarily as complex lipids like phospholipids and glycolipids, which are also involved in diverse biological signaling and processes (Cortes Ballen et al., 2024). To adapt to hypoxia, tumor cells reprogram their lipid metabolism, enhancing their utilization of fatty acids and boosting lipid production by harnessing both *de novo* lipogenesis (DNL) from glucose and the uptake of exogenous lipids to sustain growth (Taïb et al., 2019; Jin et al., 2023). Glioma cells expressed fatty acid synthase (FASN), and its expression level showed a positive correlation with the degree of tumor malignancy (Tao et al., 2013). The synthesis of fatty acids originates from the Krebs cycle, where excess citrate is converted to acetyl-CoA by ATP-citrate lyase (ACLY). The resulting acetyl-CoA is then carboxylated to malonyl-CoA by acetyl-CoA carboxylase 1 (ACC1) and subsequently catalyzed by FASN to generate long-chain fatty acids such as palmitic acid (Currie et al., 2013). Acetyl-CoA synthetase short-chain family member 2 (ACSS2) regulated the conversion of acetate to acetyl-CoA for lipid synthesis. ACSS2 was highly expressed in patients with GBM and was associated with poor overall survival (Agnihotri and Zadeh, 2016).

### 2.4 Krebs cycle

The Krebs cycle acted as a central metabolic hub in glioma cells, supporting their catabolic and anabolic needs. Its maintained activity amidst nutrient restriction and aggressive growth highlighted a key metabolic adaptability that was critical for fulfilling the biosynthetic and bioenergetic demands of malignant progression (Mashimo et al., 2014; Trejo-Solís et al., 2024). Functioning as a key regulatory enzyme in the Krebs cycle and other metabolic processes, IDH had a dual role in glioma. Its wild-type forms (IDH1 in cytoplasm, IDH2 in mitochondria) normally generated α-ketoglutarate (α-KG), but the mutant isoforms (IDH1 R132H and IDH2 R172K), found in most low-grade gliomas, aberrantly produced the oncometabolite 2-HG accompanied by the consumption of NADPH, thereby contributing to tumor development (Hu et al., 2006; Strickland and Stoll, 2017; Trejo-Solís et al., 2024). Due to IDH mutation, the production of NADPH decreased and consumption increased, which led to the levels of NADPH being significantly downregulated to influence the Redox status in gliomas (van Lith et al., 2016). On one hand, the consumption of NADPH compromised the reducing equivalents available for biosynthesis, leading to the accumulation of ROS. On the other hand, NADPH depletion might also impair the regeneration of GSH, collectively influencing antigen presentation and the function of T cells to promote tumor growth (Han S. et al., 2020; Torrisi et al., 2023). 2-HG, which accumulated to approximately 100-fold higher concentrations in patients with IDH1/2 mutations, drove gliomagenesis through multiple interconnected mechanisms (Nam et al., 2014; Bunse et al., 2018). Firstly, it competitively inhibited α-KG-dependent histone demethylases, impairing cellular differentiation (Xu et al., 2011). Secondly, it induced a unique epigenetic state known as the CpG island methylator phenotype (CIMP), characterized by widespread promoter hypermethylation that silenced tumor suppressor genes (Cortes Ballen et al., 2024). Furthermore, 2-HG promoted tumorigenesis by inhibiting the angiogenesis inhibitor endostatin, thereby enhancing glioma vascularization and growth (Liu et al., 2012). IDH-mutant gliomas exhibited a distinct metabolic profile, including the specific expression of glutamate dehydrogenase 2 (GLUD2) to generate α-KG, which was subsequently converted to 2-HG. This pathway fundamentally differentiated them from aggressive, α-KG-producing IDH-wildtype GBM, and underlay the slower progression and enhanced treatment sensitivity characteristic of the mutant subtype (Chen et al., 2014; Han S. et al., 2020).

Dysregulation of key Krebs cycle enzymes contributes to gliomagenesis. Citrate synthase (CS), catalyzing the cycle’s first and irreversible step, was significantly downregulated in GBM compared to low-grade gliomas, suggesting a role in promoting tumor aggressiveness (Fan et al., 2020). Conversely, succinate dehydrogenase (SDH/Complex II), a tumor suppressor with dual roles in the cycle and electron transport chain, was frequently mutated (Rasheed and Tarjan, 2018; Dalla Pozza et al., 2020). These SDH mutations promoted an invasive phenotype through a dual mechanism: the accumulation of succinate inactivated α-KG-dependent enzymes, leading to both the stabilization of HIF-1α and the exacerbation of epigenetic dysregulation via inhibition of histone and DNA demethylases, thereby driving tumor progression (Her and Maher, 2015; Chinopoulos and Seyfried, 2018; Garcia et al., 2021).

## 3 Characteristics of immunosenescence in the glioma microenvironment

### 3.1 Characteristics of immune cells in gliomas

Long considered an immunoprivileged zone—implying xenotransplant rejection avoidance and brain tumor immune surveillance evasion—recent studies have confirmed the presence of immune cells in the brain. While the intact BBB limits the access of immune cells to the brain under healthy conditions, it becomes disrupted in contexts like inflammation, tumorigenesis, or post-radiotherapy, permitting the influx of both lymphoid and myeloid cell populations (Bugakova et al., 2024). Tumor-associated Macrophages/microglia (TAMs), myeloid-derived suppressor cells (MDSCs), lymphocytes (CD8 cytotoxic T cells, CD4 regulatory T cells, and B cells, NK cells), and neutrophils, etc., are involved in the composition of the glioma microenvironment (Table 1) (Jayaram and Phillips, 2024).

**TABLE 1:** Characteristics of major immune cells in the glioma microenvironment.

Cell type/Subset	Key characteristics and functions	Role and regulatory mechanisms
TAMs	Origin: CNS-resident microglia + peripherally recruited macrophages Abundance: ∼30% of the TME cellular architecture Phenotype: Plastic continuum between M1 (pro-inflammatory/anti-tumor) and M2 (anti-inflammatory/pro-tumor) states Function: Regulate immune responses, tumor invasion, angiogenesis, and extracellular matrix remodeling	Pro-tumor role: • M2 TAMs secrete CCL17, IL-4, TGF-β, etc., fostering an immunosuppressive TME. • Secrete small extracellular vesicles (sEVs) that promote tumor proliferation/migration; interact with GSCs to mediate therapy resistance • Tumor cells upregulate CD47 (“do not eat me” signal) to bind SIRPα on macrophages, evading phagocytosis Anti-tumor potential: • CD169(+) macrophages secrete pro-inflammatory chemokines, recruiting T/NK cells and enhancing phagocytosis to boost anti-tumor immunity Key regulators: Genes like LAPTM4A, KCNE3, TSP1, LILRB4 promote M2 polarization; the miR-192/EGR1-HOXB9 axis promotes M1 polarization
MDSCs	Definition: Heterogeneous population of immature myeloid cells with potent immunosuppressive capacity, expanded under pathological conditions Subsets: Polymorphonuclear (PMN-MDSCs) and monocytic (M-MDSCs) Abundance: Constitutes ∼30–50% of the glioma mass	Clinical significance: Elevated in the peripheral blood of IDH-wildtype GBM patients, correlating with poorer prognosis Recruitment: Mediated by tumor-secreted factors via axes like CXCR2-CXCL5, M-CSF, IL-34 (M-MDSCs) and CCL2, CCL3, hypoxia (PMN-MDSCs) Function: Suppress T cell activity, promote tumor growth/angiogenesis, and diminish efficacy of immunotherapies Key regulators: • Pro-tumor: IL-8, integrin β1/DPP-4, exosomal miRNAs (miR-1246, miR-1298-5p) enhance MDSC differentiation/function • Anti-tumor: BATF2 inhibits glioma growth and MDSC recruitment
NK cells	Function: Innate cytotoxicity and cytokine (e.g., IFN-γ) secretion Subsets: CD56^dim^ (primarily cytotoxic) and CD56^bright^ (primarily cytokine-secreting)	• Function is modulated by inhibitory receptors (e.g., TIM3, TIGIT) • NK cell gene signatures correlate with glioma grade, IDH status, and other molecular features • Can be activated via: (1) glioma exosomal miR-1983 acting as a TLR7 ligand to trigger IFN-β production, or (2) MDA activating the IKKε/TBK1/IRF3 pathway to increase type I IFNs, boosting NK cell number and activity
T cells	Subsets: • CD8^+^ Cytotoxic T Lymphocytes (CTLs): Direct tumor killing • CD4^+^ T helper (Th) cells: Provide help for immune responses • Regulatory T cells (Tregs): Secrete TGF-β, IL-10 to mediate immunosuppression	• Gliomas are “immune-cold” with low TILs (1%–5%) • Tregs expand dramatically with progression (from ∼2.8% to >40%). Glioma-secreted complement Factor H (FH) acts as an ICOS ligand to enhance Treg survival/function • Immune checkpoints: Expression of TIM3, TIGIT on T/NK cells contributes to exhaustion and immune escape • Tertiary lymphoid structures (TLSs): Intratumoral immune aggregates associated with better prognosis, supporting localized anti-tumor immunity
B cells	Subsets: • Conventional B cells: Antibody production • Regulatory B cells (Bregs): Immunosuppressive functions • Plasma cells: Antibody-secreting effector cells	• Bregs: Can be activated by glioma-derived P1GF, subsequently inhibiting CD8^+^ T cell function • Plasma cells: Aberrantly enriched in GBM, exhibit low somatic hypermutation, and correlate with poor prognosis. Recruited to GSC niches via the CCL2-CCR2 axis. Antibodies may paradoxically promote GSC proliferation via FcγRIIA engagement

#### 3.1.1 Tumor-associated macrophages (TAMs)

Microglia, tissue-resident macrophages of the CNS that make up 10%–15% of all glial cells, are a significant innate immune component in the CNS (Lin et al., 2023). Microglia and peripheral macrophages composed TAMs in glioma, accounting for 30% of the cellular architecture in the glioma microenvironment (Song et al., 2025). In GBM, microglia tend to accumulate in the marginal zones, where they contribute to tumor invasion and the regulation of local immune responses. In contrast, peripheral macrophages are mainly clustered in the central regions and perivascular areas; via chemokines, they recruit relevant cells and participate in the formation of an immunosuppressive microenvironment (Zhao et al., 2025).

As is widely known, TAMs are divided into two phenotypes: the M1 phenotype tends to exhibit anti-tumor effects and the M2 phenotype tends to promote tumor progression. The M2 phenotype is further subdivided into M2a, M2b, M2c, and M2d (Mahajan et al., 2023). M1 and M2 macrophages are present at all stages of the tumor and regulate tumor development, invasion, infiltration, angiogenesis and immune response through the secretion and expression of pro-inflammatory such as C-X-C chemokine ligand (CXCL-5), CXCL-9, TNF-α, IL-12 or inhibitory factors like chemokine (C-C motif) ligand (CCL)-17, IL-4, transforming growth factor (TGF)-β, as well as be in a continuum of polarization states and can reciprocally transform in the glioma microenvironment (Tong et al., 2021). To better distinguish M1 and M2 phenotypes, researchers found MRC1, CD163 and CD204/MSR1 as M2-associated markers, and ISG15, CD86, CXCL9, CXCL10, IRF1 and CD40 as M1-associated markers (Cheng et al., 2025). However, macrophage polarization is a plastic and dynamic process, not a simple binary switch between discrete M1 and M2 states. Thus, the observed phenotypes likely indicate a shift toward a predominant state rather than a permanent, exclusive polarization (Ransohoff, 2016). Meanwhile, TAMs might regulate drug metabolism, interact with glioma stem cells (GSCs), facilitate microenvironmental adaptation, and remodel the extracellular matrix to mediate therapeutic resistance (Wang L. J. et al., 2022; Liu and Yu, 2025). Small extracellular vesicles (sEVs) are active players in the continuous exchange of intercellular information in the brain and have become valuable indicators of cancer progression and response to treatment (Tam and Yam, 2025). Interestingly, sEVs from resident macrophages enhanced cell proliferation in a dose-dependent way and promoted cell migration in gliomas (Chen et al., 2025). Recent research investigated that tumor necrosis might lead to tumor microenvironment (TME) restructuring and immunosuppressive TAMs accumulating in perinecrotic regions, resulting in tumor progression in GBM (Li J. B. et al., 2025). Due to the incomplete surgical excision of infiltrative glioma growth, the residual glioma cells increased anti-phagocytosis molecule CD47 binding to the macrophages signal regulatory protein alpha (SIRPα) to evade phagocytosis by macrophages (Ye L. et al., 2023). Based on single-cell transcriptome analysis, researchers found that CD169 (+) macrophages secreted pro-inflammatory chemokines, resulting in T cell and NK cell accumulation, and promoted phagocytosis of apoptotic glioma cells so that strengthening tumor-specific T cell responses (Kim et al., 2022).

Various genes could regulate the phenotype of TAMs to influence gliomas. According to single-cell RNA sequencing (scRNA-seq) analysis, results showed that a plethora of TAMs exhibited M2 polarization and increasing regulatory T cells (Tregs) differentiation in the glioma microenvironment (Zhang S. D. et al., 2025). Furthermore, Lysosomal-associated protein transmembrane 4 A (LAPTM4A) promoted M2 polarization of TAMs to strengthen cell proliferation and invasion, leading to glioma progression. Through constructing LAPTM4A-deficient glioma models, M1 macrophage phenotypes increased, immune activation was strongly and anti-programmed cell death protein-1 (PD-1) therapy became more sensitive (Geng et al., 2025). Researchers found that glioma cell proliferation, migration, and invasion were attenuated, with M1 polarization enhanced through silencing Potassium voltage-gated channel subfamily E regulatory subunit 3 (KCNE3) in macrophages. Further mechanism investigation showed that depleting KCNE3 inhibited Wnt/beta-catenin signaling, up-regulating the secretion of pro-inflammatory cytokines TNF-α, IL-6, and IL-12 (Liu S. Y. et al., 2025). In GBM, enhanced neuronal connectivity regions showed regional immunosuppression with anti-inflammatory TAMs enrichment. Knocking out Thrombospondin-1 (TSP1/Thbs1), suppressing synaptogenesis and glutamatergic neuronal hyperexcitability, enhanced the infiltration of pro-inflammatory TAMs and CD8^+^ T cells and lessened TAM-mediated T cell suppression, to regulate immune response in GBM (Nejo et al., 2025). Nuclear factor erythroid 2-related factor 1 (NFE2L1, also called Nrf1) knockdown contributed to the switch in the TAMs from M2 phenotype to M1 phenotype, resulting in suppression of glioma progression, accompanied by a significant increase of CD8^+^ T cells and anti-PD-1 therapy sensitivity (Zhang Q. et al., 2025). There was a negative correlation between microRNA (miRNA)-192 expression and glioma malignancy, whereas the expression of EGR1/HOXB9, a downstream regulator of miR-192, showed a positive correlation with malignant phenotypes. MiR-192/EGR1-HOXB9 loop could weaken glioma cell stemness, decrease the quantities of M2-phenotype TAMs, with lessen the inhibitory effect on CD8^+^ T cells through mediating immune chemokines. Interestingly, miR-192 also induced immune infiltration *via* this loop *in vivo* (Li G. W. et al., 2025). The leukocyte immunoglobulin-like receptor B4 (LILRB4), a pivotal immunoregulatory molecule implicated in cancer progression, showed a negative correlation with survival in GBM. Additionally, knockdown of LILRB4 switched M2 phenotype to M1 phenotype through STAT3/IL10 axis, which might regulate immunotherapy efficacy in gliomas (Pei et al., 2025).

#### 3.1.2 Myeloid-derived suppressor cells (MDSCs)

Myeloid cells, a pivotal part of innate immunity playing an important role in immunotherapy, differentiate into macrophages, granulocytes, and dendritic cells (DCs) in normal. However, under pathological situations, the differentiation of immature myeloid cells in disrupted, resulting in myeloid-derived suppressor cells (MDSCs) developed, proliferated and activated (Khan et al., 2020; Parizi et al., 2021). MDSCs might not only exert pivotal roles in suppressing immune responses within the TME but also regulate the progression of tumor growth, which is related to some small molecules, NK cells, cytokines, inflammatory proteins, and so on (Ge et al., 2021; Jackson et al., 2025). Granulocytic or polymorphonuclear MDSCs (PMN-MDSCs), which are similar to neutrophils in phenotype and morphology, and monocytic MDSCs (M-MDSCs), which are similar to monocytes, compose MDSCs (De Leo et al., 2021). Research data indicated that MDSCs account for approximately 30%–50% of the glioma mass (Parizi et al., 2021). In patients diagnosed with gliomas, a notable elevation of MDSCs has been observed in the peripheral circulation of those with isocitrate dehydrogenase-wildtype (IDH-WT) glioblastoma; furthermore, higher MDSC levels in this subgroup are correlated with a poorer clinical prognosis (Jackson et al., 2025). In GBM, tumor-secreted factors established chemokine gradients to recruit MDSCs to the TME to play a fundamental role. The CXCR2-CXCL5 signaling axis, M-CSF, and IL-34 mediated the accumulation and differentiation of M-MDSCs, and CCL2, CCL3, and hypoxic stress drove the infiltration of PMN-MDSCs (Takacs et al., 2024; Li Y. T. et al., 2025). Based on single-cell RNA sequencing, researchers observed upregulation of genes involved in nuclear factor of activated T cells (NFAT) signaling and cellular response to hypoxia, accompanied by a downregulation of genes responsible for mediating inflammatory responses and regulation of T cell proliferation in PMN-MDSCs. Furthermore, more CXCL2 and less CXCL3 were expressed by PMN-MDSCs in the early stage than the late stage of GBM (Yeo et al., 2022).

A subpopulation of CD4^+^ T cells producing IL-8, myeloid and tumor cells, regulated MDSCs infiltration and angiogenesis, leading to accelerated tumor growth and weakened immune checkpoint blockade (ICB) efficacy (Liu H. et al., 2023). In both mouse and human, M-MDSCs exhibited elevated expression of integrin (31 and dipeptidyl peptidase-4 (DPP-4) relative to PMN-MDSCs. This differential expression pattern was a key component of the enhanced cell adhesion signature characteristic of M-MDSCs. Functional studies demonstrated that blockade of integrin β31 not only abrogated the tumor-promoting phenotypic features of M-MDSCs but also induced significant alterations in the immune cell composition and functional profile of the TME. In parallel, phospho-Extracellular Signal-Regulated Kinase (p-ERK) signaling and their migration towards tumor cells could be inhibited through targeting DPP-4 in M-MDSCs (Bayik et al., 2022). The basic leucine zipper ATF-like transcription factor 2 (BATF2) was involved in the regulation of immune cells and anti-tumor effects, which could inhibit glioma growth and the recruitment of MDSCs, possibly related to regulating SDF-1α/CXCR4 signaling pathway (Zhang X. et al., 2021). The interaction between glioma cells and immune cells might be through the secretion of exosomes, like microRNAs. MiR-1246 was enriched in glioma-derived exosomes and exosomes isolated from the cerebrospinal fluid of glioma patients, which could mediate the differentiation and activation of MDSCs in a manner dependent on dual specificity phosphatase 3 (DUSP3)/ERK (Qiu W. et al., 2021). Meanwhile, miR-1298-5p could strengthen the immunosuppressive effects of MDSCs to promote gliomas (Qi et al., 2022). Besides, the expression levels of other cytokines like sialic acid-binding Ig-like lectin 9 (SIGLEC9), CCR2, CCR5, factor H like protein 1 (FHL-1) and Annexin A2 (ANXA2) could regulate MDSCs infiltration and accumulation (Ma et al., 2021; Zhai et al., 2021; Xu et al., 2022; Pant et al., 2024).

#### 3.1.3 Lymphocytes

Lymphocytes mainly include NK cells, T cells and B cells. Because of the low presence of tumor-infiltrating lymphocytes (TILs), gliomas are named as immune “cold tumors”, accompanied by systemic and local suppressive immune response. NK cells can recognize tumor cells and initiate a cytotoxic cascade, as well as recruit other innate and adaptive immune cells. NK cells are equipped with both stimulatory (or activation) receptors and inhibitory receptors, which enable them to distinguish between healthy cells and aberrant cells through MHC-1 receptor appearance (Read et al., 2024; Wu et al., 2025). NK cells are divided into CD56^bright^ and CD56^dim^ subsets. CD56^dim^ NK cells predominantly mediate cytotoxicity, in contrast to CD56^bright^ NK cells, which function primarily as secretors of cytokines including IFN-γ and TNF-α (Zhao et al., 2020; Zhang et al., 2024). Based on CGGA databases, the expression of NK cells gene expression was related to WHO grade, IDH1 mutation, MGMT promoter methylation, 1p19q ci-deletion, and tumor subtype. Following screening, UL16 binding proteins (ULBPs), CD70 and BH3-interacting domain death agonist (Bid) were identified to be implicated in the biology and function of NK cells (Li et al., 2022). As GBM progression, NK cells accumulated based on transcriptome analysis. Meanwhile, the proportion of CD3^+^/CD4^+^ helper T cells decreased in GBM patients, and the frequency of NK cells significantly augmented in grade 3 gliomas (Monaco et al., 2022; Lennartz et al., 2023). Glioma-released miR-1983 within exosomes, initiating an innate anti-glioma NK-mediated circuit, was demonstrated. Further research showed that miR-1983 was an endogenous TLR7 ligand; subsequently, secretion of IFN-β, stimulated by TLR7 activation and downstream signaling through MyD88-IRF5/IRF7, stimulated NK cells, leading to glioma eradication (Shah et al., 2021). In glioma models, MDA mediated IKK epsilon/TBK1/IRF3 signaling pathway, subsequently induced the increase of IFN-I, resulting in boosting CD8^+^ T cell and NK cell number and activity (Mu et al., 2023).

T cells comprise CD8^+^ cytotoxic T lymphocytes (CTLs), CD4^+^ Tregs and conventional CD4^+^ T cells, accounting for 1%–5% of gliomas. Upon binding to specific molecules, CD8^+^ T cells differentiated into CTLs capable of releasing perforin- and granzyme-containing granules and CD4^+^ T helper 1 (Th-1) cells secreted pro-inflammatory cytokines such as IL-2, TNF-α and IFN-γ, thereby promoting antitumor activity (Elguindy et al., 2024). A single-cell analysis showed that the infiltration of T cells increased during glioma progression, and the ratio of T cells was higher in recurrent GBM compared to low-grade gliomas and newly diagnosed GBM (Abdelfattah et al., 2022). Through flow cytometry and survival data analysis, it was discovered that the frequency of Th cells and CTLs was negatively correlated with glioma survival, whereas there was a positive relationship between the frequency of gamma delta-T cells and CD56 bright NK cells and survival (Vincze et al., 2024). In gliomas, high cytotoxicity tumor-infiltrating CD8 T cells were correlated with an NK cell-like signature. For example, large numbers of CD8 T cells exhibited expression of both the inhibitory CD161 receptor (KLRB1), which was inactivated in tumor-infiltrating T cells, strengthening their anti-tumor activity *in vivo* and the activating NKG2C/CD94 receptor (KLRC2 and KLRD1) (Mathewson et al., 2021). The checkpoint and inhibitory receptor HAVCR3 (TIM3) were highly expressed on and BAT 3 was lowly expressed on activated CD4^+^ and CD8^+^ T cells, together with NK cells from GBM patients, related to downregulation of CD69 and IFN gamma, which might be related to glioma immune escape (Zhang Z. et al., 2023; Ahmady et al., 2024). Through CRISPR-Cas9 knockout of TIM3, the abilities of NK cell-mediated growth inhibition of GBM cells were augmented (Morimoto et al., 2021). Another immune checkpoint receptor, TIGIT, is expressed on activated T cells and could inhibit the function of T cells and NK cells to influence GBM (Vincze et al., 2024). ITPRIPL1, CD3 epsilon-inhibitory ligand suppressing the activation of T cells, negatively correlated with CD4^+^ T cells and Th17 cells which could inhibit T cell-mediated immune responses, promoting the development of immunosuppressive TME, thereby promoting tumor progression (Zou et al., 2025).

Tregs, involved in anti-inflammatory and immune tolerance, could secrete inhibitory cytokines TGF-β and IL-10 in TME and exhaust CTLs to regulate immunosuppressive effects, and Forkhead box protein P3 (Foxp3) (+) Tregs played a significant role in the immunosuppressive TME (Xu et al., 2020; Guo et al., 2021). As glioma progression, the ratio of Tregs augmented (from 2.8% to over 40%), verifying the activating effect of GBM with respect to Tregs (Yanysheva et al., 2025). In mouse and human gliomas, tumor cells secreted complement factor H (FH), the upregulation of which was related to the existence of Tregs and the worse prognosis for glioma patients. Furthermore, FH was a kind of Inducible co-stimulator (ICOS) ligand, and the interaction between FH and this immune checkpoint molecule enhanced the survival and functional activity of Tregs, stimulated the release of TGF-β and IL-10, and concurrently inhibited T cell proliferation (Smolag et al., 2025). A recently identified subset of Tregs is T follicular regulatory (Tfr) cells, and both Tfr cells and Tregs exhibited a significant ability to suppress the proliferation of CD8^+^ T cells and their cytotoxic activity against glioma tumor cells. CXCR5 (−) Tregs traditionally showed stronger inhibitory potency on CXCR5 (−) CD8 T cells, whereas Tfr cells exhibited higher suppressive potency on CXCR5 (+) CD8 T cells (Lu et al., 2021).

Tertiary lymphoid structures (TLSs), the intratumoral immune aggregates, are suggested to regulate sustained anti-tumor immune responses. TLSs, which are ectopically formed aggregates of lymphoid and stromal cells consisting of T cell zones with antigen-presenting dendritic cells and B cell zones with germinal centers, support local immune responses through several mechanisms, such as antigen presentation to T cells and differentiation of B cells into plasma cells that secrete tumor-specific antibodies. In gliomas, some TLSs exhibited dynamic immune function features (clonal T and B cell expansion, generation of IgA^+^ and IgG^+^ plasma cells, and dendritic cell-T cell interactions), related to overall survival (Cakmak et al., 2025).

B lymphocytes producing antibodies are key mediators of humoral immunity, and tumor-infiltrating B cells play a key role in cancer immunity (Ma et al., 2024). Like Tregs, regulatory B cells (Bregs) expressed immunosuppressive surface molecules and cytokines and suppressed CD8^+^ T cell proliferation and function, thereby influencing immunotherapy response (De Domenico et al., 2025). Glioma cell-derived placenta growth factor (P1GF) induced the activation of Breg cells, subsequently inhibited the proliferation of CD8^+^ T cells and the release of perforin and granzyme B to influence gliomas (Han et al., 2014). Plasma cells, the primary effector cells of the B-lineage immune system, were aberrantly enriched within the B-cell population that infiltrated GBM. These tumor-infiltrating plasma cells exhibited low levels of somatic hypermutation and were correlated with poor prognosis. Their recruitment into GBM stem cell (GSC) niches is mediated by the CCL2-CCR2 chemokine axis. Furthermore, GSCs acquired pro-proliferative signals through FcγRIIA activation, a pathway engaged by widely used monoclonal antibody-based immune checkpoint inhibitors (Gao et al., 2025).

### 3.2 Immunosenescence leading immunosuppressive microenvironment

#### 3.2.1 Immune cells

**FIGURE 2:**
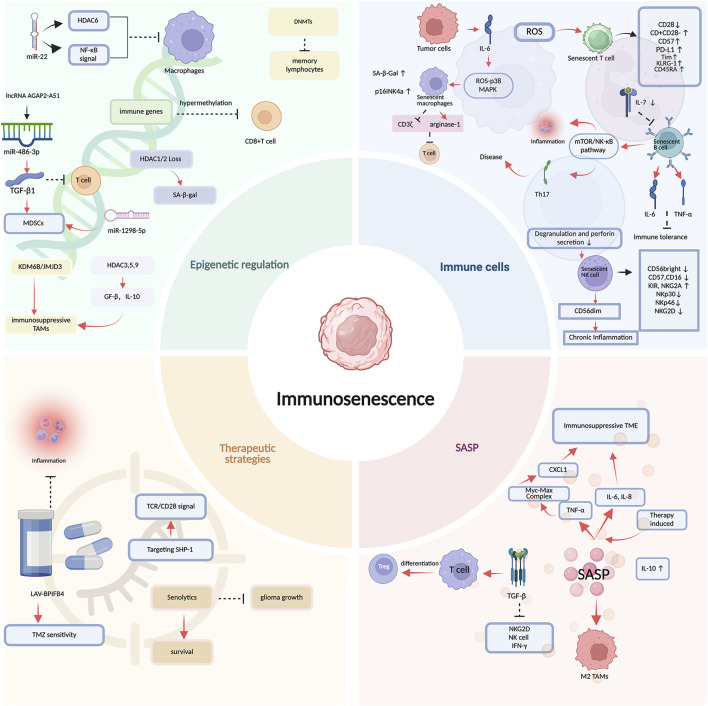
The hallmarks of immunosenescence in gliomas. The senescence of T cells, B cells, and NK cells involves alterations in the levels of molecular markers, which regulate signaling pathways to inhibit immune responses and promote chronic inflammation, among other effects. Senescent immune cells secrete SASP to influence the function of immune cells, thereby fostering the formation of an immunosuppressive microenvironment. Epigenetic changes, such as DNA methylation, histone modifications, and altered expression of non-coding RNAs, further contribute to the establishment of this immune microenvironment. Targeting the key mechanisms of immune senescence can enhance drug sensitivity, suppress inflammation, and extend survival. Red arrows: promoting or up-regulating effects; black arrows: regulating effects; black T-shaped lines: inhibitory effects. Supported by BioRender (https://app.biorender.com).

Senescent macrophages showed impaired metabolism, low-grade inflammation, reduced autophagy level, decreased phagocytosis, altered polarization tendency, altered antigen presentation, and altered infiltration and recruitment. Cellular senescence entails cell cycle arrest and lysosomal expansion. These processes are primarily governed by the p16^Ink4a^/RB and p53/p21^CIP1^ signaling pathways and are mechanistically linked to the elevated activity of senescence-associated beta-galactosidase (Figure 2) (SA-β-Gal) (Li et al., 2024; Fu and Zhou, 2025). Immunosenescence triggers signaling cascades that ultimately converge on the p53/p21^CIP1^ and p16^INK4a^/pRB pathways, which suppress the cell cycle through dynamic interactions with cyclin-dependent kinases (CDKs); therefore, p16^INK4a^ and p21^CIP1^ are applicable biomarkers for *in vivo* immunosenescence detection (Liu Z. Q. et al., 2024). Under pH 6.0, the activity of lysosomal β-galactosidase is upregulated in senescent cells, contributing to SA-β-Gal might be used as a biomarker of immunosenescence, and elevated levels of SA-β-Gal are linked to compromised immune cell function (Tsubokawa et al., 2025; Martinez-Zamudio et al., 2021). In the glioma microenvironment, the expression of SA-β-Gal on microglia and macrophages, and senescent macrophages, was related to the upregulation of SA-β-Gal, p16^INK4a^, SASP (such as IL-1α, IL-6, CXCL12, FGF, VEGF, MMPs, and TGF-β), and so on (Salam et al., 2023; Li et al., 2024). Furthermore, the partial depletion of p16 ^Ink4a^ cells led to the modulation of TAMs abundance and activity: upregulation of microglia-related pro-inflammatory genes and downregulation of macrophage-related anti-inflammatory genes (Salam et al., 2023). Glioma-derived IL-6 induced a senescent phenotype in TAMs *via* the ROS-p38 MAPK pathway. Subsequently, these senescent macrophages produced arginase-1, which suppressed CD3ζ expression and thereby dampened T-cell responses (Li et al., 2024; Shen et al., 2025).

Senescent T cells are characterized by impaired proliferation, defective signaling pathways, and a reduced ability to differentiate into effector cells (e.g., weakened differentiation of CD8^+^ T cells into cytotoxic T cells) (Nikolich-Zugich, 2014). The hallmark of senescent T cells was the decrease of costimulatory molecule CD28 which might be associated with telomerase inactivation, with upregulated CD8^+^CD28^−^senescent populations with heterogeneous roles in multiple solid and hematogenous tumors. CD8^+^CD28^−^ T cells, similar to Tregs, showed immunosuppressive effects through inducing suppressive receptors to influence Ag-presenting function. Besides, CD57 (HNK-1) is a hallmark of senescent T cells, and CD8^+^ CD28^−^ CD57^+^ populations were expanded in various pathologies, including cancer (e.g., lung cancer, myeloma), HIV, and chronic inflammatory diseases (e.g., diabetes, obesity) (Huff et al., 2019; Nga et al., 2024). Continuous T cell activation also led to the continuous shortening of telomeres and DNA damage, such as exposure to ROS, resulting in T cell senescence. GBM patients with an increased proportion of T cells showing high CD57 and low CD28 expression led to a marked reduction in overall survival (Nafe and Hattingen, 2024). In elderly GBM patients, an upregulation of immune checkpoint genes such as PD-1 ligand-1(PD-L1) and CD80 in T cells drove an immunosuppressive state, which was manifested by a diminished population of cytotoxic CD8^+^ T cells and an expanded population of exhausted CD4^+^ T cells (Wu et al., 2023). Besides, elevated expression of other immune biomarkers, including Tim-3, killer cell lectin-like receptor subfamily G member 1 (KLRG-1), and CD45RA, serves as a well-established marker for the T-cell senescence phenotype (Liu Z. et al., 2023).

Senescent NK cells can be characterized by age-related or pathology-induced changes in their functional capabilities and phenotypic traits. An imbalance in NK cell subset proportions diminished cytotoxic activity. During aging, CD56^bright^ NK cells decreased, and CD56^dim^ NK cells underwent continued differentiation, progressively augmenting the expression of CD57, CD16, inhibitory receptor KIR and NKG2A, and downregulation of the activating receptor NKp30, NKp46, and NKG2D, which collectively contributed to the reduction in NK cell cytotoxic function (Qiu et al., 2025). Besides, a decline in degranulation and perforin secretion was accompanied by a shift of NK cells toward a CD56dim subset characterized by enhanced production of pro-inflammatory cytokines, thereby potentially fueling chronic inflammation (Fu et al., 2025).

Age-related changes in B cell composition are a major cause of weakened antibody responses to vaccination and infection in the elderly. Declining levels of IL-7, a cytokine critical for pre-B cell survival, severely constrained B cell output. This was compounded by reduced RAG expression in precursor B cells, which diminished BCR rearrangement, limited diversity, and ultimately led to impaired antigen recognition and humoral immunity (Labrie et al., 2004). Immunosenescence promoted autoimmune progression via age-associated B cells (ABCs). These senescence-dysfunctional ABCs adopted a proinflammatory state, secreting IL-6 and TNF-α while producing auto-antibodies, thereby disrupting immune tolerance. Additionally, they exacerbated chronic inflammation by activating mTOR/NF-κB signaling, which promoted the pathological activation and tissue infiltration of cells like Th17, ultimately accelerating disease (Xiao et al., 2025).

The collective senescence of various immune cells drives tumor progression by fostering an immunosuppressive microenvironment.

#### 3.2.2 SASP

SASP, the core hallmarks of immunosenescence, encompasses a diverse array of factors, including pro-inflammatory cytokines, chemokines, growth factors, ROS, angiogenic factors, and proteases. These components collectively foster a pro-tumorigenic cytokine microenvironment (Qiu et al., 2025). Additionally, the SASP drives tumor progression by enhancing growth, invasion, and immune evasion, which collectively foster an immunosuppressive TME (Fu et al., 2025). The immunosuppressive factors produced by senescent cells, including TGF-β and IL-10, inhibited NK cell function. Specifically, TGF-β downregulated the expression of activating receptors such as NKG2D on NK cells and impaired their production of IFN-γ. In addition to directly suppressing NK cells, TGF-β also promoted the differentiation of regulatory Tregs from effector T cells, further dampening anti-tumor immunity (Gergues et al., 2025). SASP activation was a hallmark of malignant progression in IDH-WT glioma. Patients with high SASP scores exhibited poorer survival, accompanied by a dysregulated immune response and abundant infiltration of M2-phenotype TAMs. Notably, TAMs themselves were a key cellular source of high-level SASP secretion (Liu Y. et al., 2025).

Therapy-induced senescence by temozolomide and radiotherapy, mediated by the DNA damage response and p21^CIP1^, activated the NF-κB pathway and was characterized by the secretion of SASP components such as IL-6 and IL-8. In xenograft mouse models, the co-injection of irradiated, senescent primary GBM cells with non-irradiated GBM cells promoted larger and more aggressive tumors compared to the injection of non-irradiated cells alone (Carreno et al., 2021). In addition, radiation-induced senescent astrocytes released TNF-α combing with TNFR1 on the surface of GBM cells to activate the downstream Myc-Max transcription factor complex. Subsequently, CXCL1 transcription promoted by Myc further recruited immunosuppressive cells into glioma TME. Besides, factors within the SASP, such as IL-6 and G-CSF, could activate inflammatory responses in neighboring normal cells through paracrine signaling, thereby amplifying the overall immunosuppressive signaling within the microenvironment (Ji et al., 2024).

### 3.3 The critical role of epigenetic regulation

Epigenetics involves heritable modifications that regulate gene expression without changing the DNA sequence (Liu Y. et al., 2024). Epigenetic dysregulation—through alterations in DNA methylation, histone modifications, and non-coding RNAs—represents a fundamental hallmark of immunosenescence (Liu Z. Q. et al., 2024).

#### 3.3.1 DNA methylation

DNA methylation is mediated by DNA methyltransferases (DNMTs), which transfer a methyl group (-CH3) from S-adenosyl-L-methionine to the C5 position of cytosine, forming 5-methylcytosine (5mC) and leading to gene silencing. “*De novo*” methylation by DNMT3 was a critical determinant in guiding post-response T cell fate towards memory versus terminal effector lineages, and inhibition of DNMT3 promoted the formation of memory lymphocytes. Furthermore, the more marked age-related functional decline in CD8^+^ T cells compared to CD4^+^ T cells correlated with hypermethylation of pivotal immune genes, thereby limiting their functional adaptability (Rousseau et al., 2025). In CD4^+^CD28^−^ T cells, loss of DNA methylation primarily occurred outside of cytosine phosphate guanine (CpG) islands, related to protein tyrosine kinase genes, the CD3 complex, and upregulated expression of B cell lymphoma/leukemia type 2 (BCL-2), TYRO protein tyrosine kinase-binding protein (TYROBP), and GzmB (Nga et al., 2024). Variations in DNA methylation patterns might shape the immune landscape of the glioma microenvironment, with potential implications for the response to PD-1/PD-L1 inhibitor therapy (Luo et al., 2022).

#### 3.3.2 Histone modifications

Histone modification is a fundamental epigenetic mechanism that includes a diverse array of chemical alterations (Wang and Yan, 2025). Among these, histone acetylation, phosphorylation, and heterochromatin accumulation show an age-associated increase (Liu Z. et al., 2023). Senescence in lymphocytes, specifically in CD8^+^ CD28^−^ T cells and NKT-like cells, might be correlated with a loss of histone deacetylase 2 (HDAC2), and loss of HDAC1/2 function in mouse podocytes led to the acquisition of a senescent phenotype, characterized by enhanced SA-β-gal activity and lipofuscin deposition (Xiao et al., 2025). In human GBM, high expression of the histone 3 lysine 27 demethylase KDM6B/JMJD3 in intratumoral myeloid cells (including TAMs) promoted an immunosuppressive phenotype, which in turn fuels tumor progression (Riyas Mohamed and Yaqinuddin, 2025). Additionally, histone modification might regulate immune cell infiltration and TGF-β and IL-6 to accelerate the progression of LGG (Wang and Yan, 2025). The upregulation of HDAC3, 5, and 9 in IL-4-induced M2-pheotype TAM during GBM enhanced the secretion of TGF-β and IL-10, ultimately potentiating the immunosuppressive activity of the tumor cells (Chen N. et al., 2022).

#### 3.3.3 Non-coding RNAs

Non-Coding RNAs, such as Long non-coding RNAs (lncRNAs) and miRNAs, function as central epigenetic regulators of immune escape. For instance, the lncRNA AGAP2-AS1 promoted an immunosuppressive microenvironment by sequestering miR-486-3p, which led to upregulated TGF-β1 secretion, subsequent activation of myeloid-derived suppressor cells (MDSCs), and diminished T-cell infiltration (Riyas Mohamed and Yaqinuddin, 2025). Low expression of miR-22 lessened the phagocytic capacity of macrophages, leading to weakened phagocytosis, antigen presentation, and inefficient T cell activation, primarily through targeting HDAC6 and modulating NF-κB signaling (Tu et al., 2022). Another miR-1298-5p was found to strengthen the immunosuppressive effects of MDSCs, resulting in glioma progression (Qi et al., 2022).

## 4 Interaction between immunosenescence and metabolic reprogramming

Metabolic reprogramming not only directly influences tumor cell proliferation, but the tumor microenvironment it shapes may profoundly influence the function and fate of infiltrating immune cells. In the meantime, immunosenescence may reshape the metabolic microenvironment to influence tumor progression. Thus, neither immunosenescence nor metabolic reprogramming exists in isolation; rather, they interact with each other and collectively influence tumorigenesis and progression.

### 4.1 Metabolites may drive immune senescence

**FIGURE 3:**
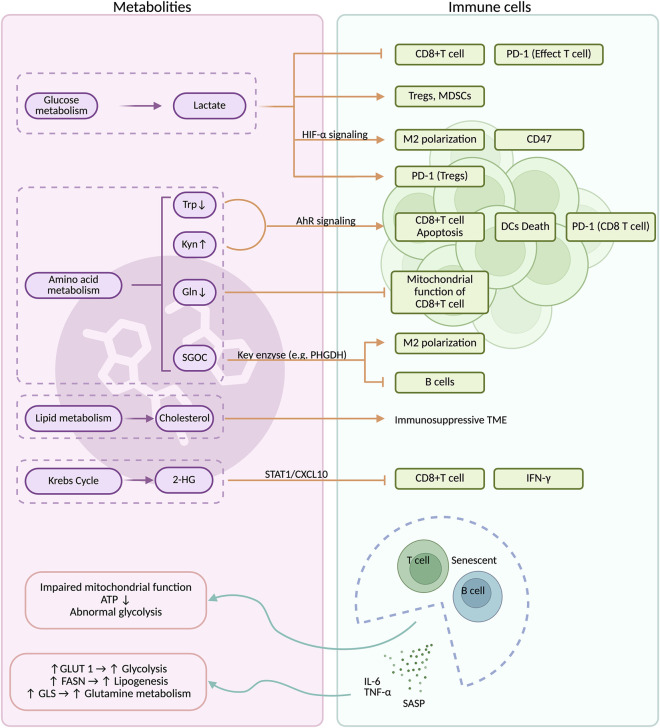
The potential interaction between metabolites and immunosenescence. Metabolic reprogramming can regulate immune cell function by modulating multiple signaling pathways and factors. Conversely, senescent immune cells also influence the metabolic microenvironment by regulating key factors involved in metabolism. Orange arrows: promoting or up-regulating effects; green arrows: regulating effects; orange T-shaped lines: inhibitory effects. Supported by BioRender (https://app.biorender.com).

The efficacy of chemotherapy and immunotherapy might be influenced by metabolites through immunomodulation (Fan et al., 2024). Through key alterations in pathways such as glycolysis, lipid, and amino acid metabolism, glioma cells create an immunosuppressive tumor immune microenvironment. They release abnormal metabolites that cripple the function of antitumor immune cells, thereby facilitating sustained tumor growth by evading immune surveillance (Figure 3) (Xia et al., 2021).

#### 4.1.1 Glucose metabolism

While various immune cells exhibited heightened glycolysis and lactate production, the resultant lactate accumulation in the tumor microenvironment impaired immune surveillance, protecting tumor cells from NK cells, neutrophils, and MDSCs that infiltrate hypoxic areas (Husain et al., 2013). Lactate in the tumor microenvironment drove immunosuppression by directly inhibiting CD8^+^ T cell function and migration while promoting the accumulation of Tregs and MDSCs, thereby facilitating tumor immune escape (Wang et al., 2023; Yang et al., 2024; Vijayanathan and Ho, 2025).

Within the glycolytic tumor microenvironment, lactate sustained a persistent immunosuppressive state by promoting HIF-1α stabilization in MDSCs, which exacerbated hypoxia, acidified the milieu, and potently inhibited T cell activity (Husain et al., 2013; Noman et al., 2014; Johnston et al., 2019). Furthermore, lactate differentially regulated immune checkpoints by upregulating PD-1 expression on Tregs while suppressing it on effector T cells, a mechanism demonstrated by Kumagai et al. to facilitate tumor immune evasion (Kumagai et al., 2022). Given the crucial role of the mTOR signaling pathway in driving the metabolic switch of T cells from oxidative phosphorylation to glycolysis, a therapeutic strategy that combined mTOR inhibitors with glycolysis inhibitors might disrupt this metabolic reprogramming and enhance anti-tumor efficacy (Abdel-Wahab et al., 2019).

Lactate produced by GBM cells enhanced M2-pheotypes polarization of TAMs and stimulated tumor angiogenesis (Wang et al., 2024). A potential mechanism was that lactate could upregulate the expression of vascular endothelial growth factor (VEGF) and arginase 1 (ARG1) genes through an HIF-1α-mediated pathway, thereby facilitating M2 polarization (Colegio et al., 2014). In the GBM immune microenvironment, Hexokinase 3 (HK3) facilitated immune cell infiltration, with its expression level correlating positively with tumor grade (Li S. et al., 2023; Cortes Ballen et al., 2024). In addition, lactate could activate the HIF-1α pathway, promote the secretion of immunosuppressive factors, induce histone lactylation, and upregulate “do not eat me” signals such as CD47, thereby inhibiting the functions of myeloid cells and NK cells (Keith et al., 2011; Venneti and Thompson, 2017). Separately, TAMs engaged in metabolic crosstalk by releasing IL-1β, which phosphorylated and activated GA3PDH in glioma cells, thereby inducing a glycolytic and proliferative state (Lu et al., 2020). Meanwhile, HIF-1α played a crucial role in orchestrating this metabolic reprogramming in both GBM cells and TAMs (Li Y. et al., 2023).

#### 4.1.2 Amino acid metabolism

Reprogrammed glutamine metabolism fostered competitive interactions between GBM and immune cells. This metabolic competition impaired antitumor immune responses, thereby reinforcing the immunosuppressive features of the tumor microenvironment. Specifically, metabolic predation by GBM restricted glutamine availability to immune cells, compromising their functions and facilitating tumor immune evasion. For instance, glutamine deprivation could impair CD8^+^ T cell function by inducing mitochondrial damage (Wang W. et al., 2022).

The catabolism of Kyn by IDO/TDO suppressed antitumor immunity *via* a multifaceted mechanism involving Trp depletion and Kyn accumulation. These effects collectively inhibited immune cell function, induced T cell apoptosis and DCs death, promoted Tregs and TAMs recruitment, and activated an AhR-driven positive feedback loop that upregulated PD-1 on CD8^+^ T cells, thereby establishing a profoundly immunosuppressive microenvironment (Zeitler and Murray, 2023; Srivastava et al., 2025). Metabolic reprogramming of specific amino acids within the GBM microenvironment critically orchestrated immunosuppression. The upregulation of serine-glycine-one-carbon pathway enzymes, such as PHGDH in endothelial cells and PSPH/SHMT1 in tumor cells, impaired antitumor T cell infiltration and altered immune cell landscapes by promoting M2 macrophage and resting NK cell recruitment while reducing B cells; notably, PHGDH inhibition could overcome resistance to CAR-T cell therapy (Chen S. et al., 2022; Zhang D. et al., 2023). In parallel, arginine metabolism further suppressed immunity, as tumor-infiltrating dendritic cells (TIDCs) depleted local L-arginine via arginase, directly impairing T cell function (Norian et al., 2009).

#### 4.1.3 Lipid metabolism

The nuclear receptor NR4A2, activated in microglia under oxidative stress, facilitates GBM immune evasion by coordinately upregulating lipogenic pathways to support tumor growth and suppressing MHC-I and antigen presentation machinery to impair immune recognition, thereby creating an immunosuppressive microenvironment (Ye Z. et al., 2023; Cortes Ballen et al., 2024). Targeting squalene monooxygenase (SQLE), a key regulator of cholesterol metabolism in TAMs, represented a promising strategy for disrupting the tumor microenvironment. Pharmacological inhibition of SQLE with terbinafine could alleviate the immunosuppressive tumor microenvironment and enhance the efficacy of immune checkpoint blockade therapy (Ye Z. et al., 2023).

#### 4.1.4 Krebs cycle

2-HG, key regulators in the immune system, reduced the level of signal transducer and activator of transcription 1 (STAT1), CXCL10, and the key factor in recruiting CD8^+^T cells. Consequently, 2-HG decreased CD8^+^T cell infiltration in glioma tumor tissues of syngeneic mice (Reiter-Brennan et al., 2018). Elevated 2-HG production induced by IDH mutations could regulate TAMs and other immune cells, forming an immunosuppressive microenvironment (Virtuoso et al., 2021). Patients with IDH1 and IDH2 mutations exhibited reduced CD8^+^ T cell cytotoxicity and attenuated interferon-γ (IFN-γ) signaling, both of which contributed to the immunosuppressive tumor microenvironment (Notarangelo et al., 2022).

### 4.2 Immunosenescence may remodel the metabolic microenvironment

By disrupting fundamental nutrient metabolism (glucose, lipids, amino acids) and NAD^+^ homeostasis in immune cells, immunosenescence promoted inflammation and accelerated its own progression. Meanwhile, reduced glycolysis and aberrant mitochondrial energetics jointly impaired T and B cell activation in the process of immunosenescence (Liu Z. Q. et al., 2024). Aging immune cells activated STAT3 and NF-κB pathways and secreted pro-inflammatory SASP such as IL-6, IL-8, TNF-α and CXCL1 to increase GLUT1, FASN and GLS, resulting in enhanced glycolysis, lipogenesis and glutamine dependency (Zhu et al., 2025).

Senescent T cells exhibited markedly reduced lactate dehydrogenase (LDH) activity and proliferative capacity. Specifically, senescent naive CD4^+^ T cells show a decreased extracellular acidification rate (ECAR), accompanied by reductions in metabolites associated with glycolysis and the pentose phosphate pathway. These cells also developed smaller mitochondria with impaired respiratory function and diminished ATP production. This mitochondrial dysfunction might stem from a defect in one-carbon metabolism, which disrupted the biosynthesis of purines and thymidine—essential for T cell proliferation and survival—and consequently impaired effector T cell differentiation (Han et al., 2023). In senescent T cells, AMPK was activated by the low expression of adenosine triphosphate and endogenous DNA damage, which induced constitutive expression of p38. Besides, glucose deprivation and genotoxic stress led to p38 activation, resulting in reduced telomerase activity and inhibited T cell proliferation (Nga et al., 2024). Furthermore, due to constitutive PD-1 signaling in effector CD8^+^ T cells profoundly altering energy metabolism, the T Cell Receptor (TCR) was unresponsive (Fukushima et al., 2018). Senescent B cells underwent a metabolic shift toward glycolysis, leading to substantial lactate production. This lactate, exported *via* the solute carrier family 5 member 12 (SLC5A12) transporter, promotes an inflammatory phenotype characterized by the secretion of SASP factors and autoantibodies (e.g., anti-dsDNA), and further drives the polarization of CD4^+^ T cells into pro-inflammatory subsets (Llibre et al., 2025). Similarly, senescent B cell subsets displayed increased mitochondrial mass and mitochondrial reactive oxygen species (mtROS), contributing to compromised energy production and abnormal one-carbon metabolism, ultimately impairing the activation of B cells and production of antibody (Goyani et al., 2024).

Given the complexity of the glioma microenvironment, further investigations are warranted to elucidate the impact of immunosenescence on the tumor’s metabolic microenvironment.

## 5 Therapeutic strategies to target immunosenescence and metabolism

### 5.1 Targeting metabolic reprogramming

#### 5.1.1 Glycolysis and the PPP

Miyai et al. demonstrated that the H3.3K27M histone acetylation signal induced the expression of GLUT-1 and was associated with enhanced aerobic glycolysis and invasiveness in gliomas (Miyai et al., 2021). Furthermore, inhibiting GLUT-1 expression by silencing the tubulin isotype TUBB4 reduced the proliferation and formation of glioma spheroids (Guda et al., 2019). Glutor, a piperazine derivative, simultaneously targeted GLUT-1, GLUT-2, and GLUT-3 to more comprehensively inhibit tumor glycolysis (Reckzeh et al., 2019). 2-Deoxy-D-glucose (2-DG), a glucose analog, acted as a competitive inhibitor of hexokinase; the phosphorylated form of 2-DG could suppress glycolysis and inhibit GBM growth (Table 2) (Pistollato et al., 2010). Through activation of AMP-activated protein kinase, methylene blue treatment elevated the oxygen consumption rate (OCR) in GBM cell lines, while concurrently reducing the extracellular acidification rate (ECAR) and lactate production to suppress glioma proliferation (Poteet et al., 2013). Studies have shown that co-treatment with the PFKFB3 inhibitor 3PO and the anti-VEGF monoclonal antibody bevacizumab effectively reduced cell proliferation and induced apoptosis *in vitro*, while also delaying tumor growth and extending survival *in vivo* (Zhang et al., 2020)*.* R406, a spleen tyrosine kinase (Syk) inhibitor, shifted the metabolic profile of glioma cells from glycolysis toward OXPHOS, leading to increased apoptosis (Sun et al., 2019). Furthermore, acriflavine (ACF), a HIF-1α inhibitor, significantly prolonged the survival of orthotopic GBM models (Cortes Ballen et al., 2024). Targeting these pathways with agents such as dehydroepiandrosterone (DHEA, a G6PD inhibitor) or other non-specific inhibitors (e.g., genistein or imatinib mesylate) might effectively reverse PPP reprogramming in GBM (Cortes Ballen et al., 2024).

**TABLE 2:** Overview of glioma metabolic reprogramming and targeted therapeutic strategies.

Metabolic pathway	Key alterations and biological impact	Representative therapeutic strategies and targets
Glucose metabolism	Warburg effect: Aerobic glycolysis producing lactate, acidifying the microenvironment and promoting immunosuppression (Venneti and Thompson, 2017) Pentose phosphate pathway: Support nucleotide synthesis and oxidative stress resistance (Langbein et al., 2006) GLUT overexpression: High levels of GLUT-1 and GLUT-3 are associated with poor patient prognosis (Libby et al., 2021; Chamarthy and Mekala, 2023) Key enzymes: HK2, PFKFB3, and LDHA serve as potential therapeutic targets (Wolf et al., 2011a; Yang et al., 2024; Daniele et al., 2015)	GLUT inhibitors: Glutor (targets GLUT-1/2/3) (Reckzeh et al., 2019); GLUT-1 suppression via TUBB4 silencing (Guda et al., 2019) HK2 inhibitor: 2-Deoxy-D-glucose (2-DG) (Pistollato et al., 2010) PFKFB3 inhibitor: 3PO (combined with bevacizumab) (Zhang et al., 2020) HIF-1α inhibitor: Acriflavine (ACF) (Cortes Ballen et al., 2024
Amino acid metabolism	Glutamine addiction: Serves as a carbon/nitrogen source for the TCA cycle and redox homeostasis (Pavlova and Thompson, 2016) Tryptophan metabolism: The IDO1/TDO2-mediated kynurenine pathway drives immunosuppression) (Ozawa et al., 2020; Zhong et al., 2022) Methionine/Cysteine restriction: Reduce glutathione levels and induce ferroptosis (Upadhyayula et al., 2023)	Glutamine antagonist: JHU-083 (Yamashita et al., 2021) IDO1/TDO2 inhibitor: AT-0174 (synergizes with temozolomide) (Bickerdike et al., 2024) BCAT1 inhibitors: Curcumin, Gabapentin (Panosyan et al., 2017; Grankvist et al., 2018; Fhu and Ali, 2020) Arginine depletion: Potentiates cytotoxicity of CDK inhibitors (Riess et al., 2022)
Lipid metabolism	Enhanced *de* *novo* synthesis: Upregulation of key enzymes like ACLY, ACC1, and FASN correlates with tumor malignancy (Zaidi et al., 2012; Tao et al., 2023) Fatty acid uptake: FABP7-mediated fatty acid oxidation supports tumor growth and invasion (De Rosa et al., 2012)	ACLY inhibitors: Hydroxycitrate, SB-204990 (Zaidi et al., 2012) ACC inhibitor: Soraphen (Corominas-Faja et al., 2014) FASN inhibitor: TVB-2640 (combined with bevacizumab, achieving 56% ORR clinically) (Kelly et al., 2023) FABP7 inhibition: Suppresses GBM growth and invasiveness (Hoang-Minh et al., 2018)
Krebs cycle	IDH mutations: Production of the oncometabolite 2-hydroxyglutarate (2-HG), leading to epigenetic dysregulation and immunosuppression (Bunse et al., 2018; Notarangelo et al., 2022) Enzyme dysregulation: SDH mutations cause succinate accumulation, stabilizing HIF-1α(Her and Maher, 2015)	IDH1/2 inhibitors: Ivosidenib (FDA-approved), Vorasidenib (Scott et al., 2021; Tejera et al., 2020) Multi-enzyme inhibitor: Devimistat, reduces metabolites and induces cell death (Nguyen et al., 2022; Anderson et al., 2023) Glutaminase inhibitor: CB-839 (De Los Santos-Jiménez et al., 2023)
Nucleotide metabolism	Upregulated *de* *novo* synthesis: Meets the demand for DNA/RNA synthesis during rapid proliferation (Cortes Ballen et al., 2024)	CAD inhibitor: PALA (targets pyrimidine synthesis) (Raizer et al., 2010) DHODH inhibitor: BAY2402234 (Spina et al., 2022) Combinatorial strategy: Concurrent inhibition of *de novo* and salvage pathways effectively suppresses tumor progression (Laks et al., 2016)

#### 5.1.2 Amino acid metabolism

GLT1 overexpression inhibited glioma proliferation by enhancing glutamate uptake, while the glutamine antagonist JHU-083 suppressed growth and disrupted mTOR signaling—an effect synergistically enhanced when combined with L-asparaginase to deplete both glutamine and asparagine (Yamashita et al., 2021). Complementary approaches included inhibiting glutamine synthetase (GS) with actinomycin D or 5-azacytidine to reduce proliferation, and combining the GLUD1 inhibitor R162 with docetaxel to impede tumor growth *in vitro* and *in vivo* (Dranoff et al., 1985; Wang Q. et al., 2022). Furthermore, targeting branched-chain amino acid metabolism *via* Branched-chain amino acid transaminase 1 (BCAT1) inhibition with curcumin or gabapentin disrupted nucleotide synthesis and redox balance, ultimately suppressing proliferation and inducing apoptosis (Panosyan et al., 2017; Grankvist et al., 2018; Fhu and Ali, 2020).

AT-0174, a dual IDO1/TDO2 inhibitor, improved the TMZ response and prolonged survival in mouse glioma models, with upregulation of CD8^+^T cells and downregulation of CD4^+^Tregs infiltration (Bickerdike et al., 2024). Riess et al. found that arginine depletion could potentiate the cytotoxic effects of cyclin-dependent kinase (CDK) inhibitors on GBM cells by impairing mitochondrial metabolism, inducing autophagy, and blocking the DNA damage response (Riess et al., 2022). Restricting dietary cysteine and methionine improved survival in glioma-bearing mice by reducing tumor cell glutathione and triggering ferroptosis (Upadhyayula et al., 2023).

#### 5.1.3 Lipid metabolism

Inhibition of key enzymes in fatty acid synthesis presents a promising strategy against cancer. ACLY inhibitors (e.g., hydroxycitrate, SB-204990) suppressed tumor growth by disrupting acetyl-CoA production (Zaidi et al., 2012), while the polyketide soraphen specifically targeted acetyl-CoA carboxylase alpha (ACACA) (Corominas-Faja et al., 2014). Furthermore, targeting fatty acid uptake and oxidation *via* FABP7 inhibition also curbed GBM growth and invasiveness (De Rosa et al., 2012; Hoang-Minh et al., 2018). In the clinical setting, this approach had been validated by the combination of the FASN inhibitor TVB-2640 with bevacizumab, which achieved a 56% overall response rate (ORR), including complete regression in 17% of patients (Kelly et al., 2023).

#### 5.1.4 Krebs cycle

While IDH inhibition demonstrated primary efficacy in low-grade gliomas, its applicability was being explored in GBM. Pharmacologic inhibitors like ivosidenib and vorasidenib effectively penetrated the CNS and suppressed the 2-HG in IDH1-mutant GBM models (Scott et al., 2021; Tejera et al., 2020). Ivosidenib had been approved by the FDA as a standard treatment for recurrent IDH1-mutant grade 2 gliomas. Beyond IDH, additional therapeutic targets related to the Krebs cycle in GBM include glutaminase, which catalyzed the conversion of glutamine to glutamate and supplied α-KG for the Krebs cycle to regulate glioma progression (De Los Santos-Jiménez et al., 2023). Besides, devimistat could decrease the metabolites of the Krebs cycle that induced cell death, and inhibited the proliferation of GBM cells (Nguyen et al., 2022; Anderson et al., 2023).

#### 5.1.5 Nucleotide metabolism

GBM cells exhibited upregulated *de novo* nucleotide synthesis to meet the demands of rapid proliferation (Cortes Ballen et al., 2024). AMP-activated protein kinase (AMPK) served as a pivotal regulator in this process, and its dysregulation in GBM could perturb nucleotide synthesis by modulating enzymes such as ribonucleotide reductase (RR) and phosphofructokinase (PFK), thereby influencing glioma progression (Han W. et al., 2020). Phosphoinositide 3-kinase-related kinase (PIKK) inhibitors, such as ceralasertib, could inhibit *de novo* purine synthesis in GBM cells (Wang W. et al., 2021). Targeting purine metabolism can regulate mitochondrial dynamics, thereby enhancing the sensitivity of glioma cells to TMZ (D’Aprile et al., 2025). CAD inhibitors are a class of small molecules that target the multi-enzyme complex carbamoyl-phosphate synthetase 2, aspartate transcarbamylase, and dihydroorotase; examples such as PALA (N-phosphonacetyl-L-aspartate) represented a potential strategy for inhibiting pyrimidine synthesis in GBMs (Raizer et al., 2010). Similarly, targeting the mitochondrial enzyme Dihydroorotate dehydrogenase (DHODH) with agents like BAY2402234 has demonstrated significant anti-tumor efficacy in GBM xenograft models (Spina et al., 2022). Furthermore, a synergistic approach that concurrently inhibited both the *de novo* and salvage nucleotide synthesis pathways had been shown to effectively suppress GBM progression, highlighting the promise of combinatorial metabolic inhibition (Laks et al., 2016).

Targeting the multifaceted metabolic reprogramming in gliomas through single or combinatorial inhibition of key pathways in glucose, amino acid, lipid, Krebs cycle, and nucleotide metabolism represents a promising therapeutic strategy.

### 5.2 Reversing immunosenescence

Since immunosenscence inhibited immune responses in the TME, reducing the senescence of immune cells might improve the efficacy of glioma therapy. The longevity-associated variant (LAV) of the bactericidal/permeability-increasing fold-containing family B member 4 (BPIFB4) improved age-related immune dysfunction and balanced low-grade inflammation in the elderly. LAV-BPIFB4 exerted multiple effects, including the remodeling of the senescent phenotype in GBM cells, enhancement of temozolomide sensitivity, and selective reduction of T cell senescence (Puca et al., 2022). Restoring CD28 expression in human T cells countered replicative senescence by enhancing telomerase activity and proliferative potential. Furthermore, pharmacological inhibition of SRC homology 2 domain-containing phosphatase-1 (SHP-1) improved T cell function in the elderly by augmenting TCR/CD28 signaling (Huff et al., 2019). The elimination of senescent astrocytes using senolytics led to marked inhibition of glioma growth and prolonged survival in mice (Fletcher-Sananikone et al., 2021).

Targeting SASP also might strengthen immune responses. Administration of an IL-7/GM-CSF fusion vaccine to aged GBM-bearing mice triggered thymic regeneration and sustained anti-tumor immunity. This was accompanied by elevated levels of the pro-inflammatory cytokine IL-1β based on global cytokine analysis, driving dendritic cell hyperactivation and leading to an expanded brain-infiltrating T cell population dominated by long-term Th17 effector memory cells (Shireman et al., 2023). Genetic ablation or pharmacological inhibition of IL-6 might moderately increase the infiltration of T cells in GBM and prolong mouse survival (Yang et al., 2021). Administration of a TGF-β2-targeting vaccine augmented adaptive and innate immunity by expanding IFN-γ-producing CD4^+^ and CD8^+^ T cells and activating NK cells, which was associated with elevated activation markers (CD69, NKG2D) and reduced immunosuppressive signals (TGF-β2, PD-1) (Tu et al., 2023). Through a targeted screen, researchers identified a potential SASP inhibitor that markedly suppressed the proliferation and invasion of GBM cells induced by SASP-secreting senescent TAMs. This compound also reduced M2 macrophage polarization and enhanced the efficacy of PD-1 blockade (Liu Y. et al., 2025).

Given the pivotal role of epigenetic dysregulation in immunosenescence, targeting epigenetic mechanisms may represent a strategy to reverse the immunosuppressive state and enhance immune responses. In addition to promoting the expression of the cancer-testis antigen NY-ESO-1 to effectively sensitize glioma cells to T-cell-mediated lysis, DNA methyltransferase inhibitors (DNMTis) also upregulated the expression of MHC-Ⅰ in glioma cells, resulting in enhanced antigen presentation and immune recognition, an effect shared with histone deacetylase inhibitors (HDACis) (Riyas Mohamed and Yaqinuddin, 2025). Besides, HDACis could regulate the efficacy of anti-PD-1 inhibitors against gliomas through increasing PD-L1 expression and immune cytokines and T cell infiltration (Li S. et al., 2025). Targeting miRNAs could regulate Signal transducer and activator of transcription 3 (STAT3) activity and expression to directly or recruiting NK cells suppress M2-phenotype TAM polarization to inhibit gliomas, with a decrease of TGF-β1 and IL-6 (Chen N. et al., 2022).

Currently, relevant clinical trials are underway across various diseases. By employing other therapeutic agents such as miRNA mimics, the regenerative capacity of T cells or B cells can be restored or re-established, thereby enhancing the immune response (Fu et al., 2025). Meanwhile, Clinical trials targeting PD-1 (NCT03718767) and combining HDAC inhibitors with radiotherapy and chemotherapy (NCT03426891) are also underway in gliomas, suggesting that targeting specific molecules involved in immune senescence may represent novel therapeutic approaches. However, owing to the considerable variability among gliomas and the scarcity of relevant clinical trials, further research is required to develop corresponding drugs.

As a result, targeting immunosenescence could be a novel and promising approach in exploring treatments for gliomas.

## 6 Discussion and therapeutic perspectives

Gliomas represent one of the most frequent primary neoplasms of the central nervous system, comprising approximately 30% of all primary brain tumors and 80% of malignant brain tumors. The current therapeutic arsenal is primarily based on surgical intervention, radiation therapy, and chemotherapy, supplemented by the developing fields of targeted treatment and immunotherapy (Weller et al., 2024). However, the poor prognosis, high recurrence rates, and negative immunotherapy results necessitate the urgent exploration of new treatments.

Metabolic reprogramming, a hallmark of gliomas, enables not only rapid tumor cell proliferation but also immune evasion by remodeling the tumor microenvironment. Glioma cells employ metabolic reprogramming as a dual-purpose strategy. Beyond meeting biosynthetic and bioenergetic demands via the Warburg effect, glutamine dependency, and enhanced lipid synthesis, these alterations actively shape an immunosuppressive niche. By competing for critical nutrients like glucose and glutamine, and by accumulating oncometabolites such as lactate, 2-HG, and kynurenine, tumor cells directly impair the function of cytotoxic CD8^+^ T cells and NK cells, while simultaneously promoting the expansion and activation of immunosuppressive entities, including Tregs, M2-polarized TAMs, and MDSCs (Qiu R. Z. et al., 2021). This metabolic hijacking of the immune landscape suggests that targeting tumor metabolism is not merely a strategy to starve cancer cells but a viable approach to reprogram the TME. The promising preclinical and emerging clinical evidence for inhibitors against key metabolic nodes like GLUTs, IDH, FASN, and DHODH provides a compelling rationale for this approach, potentially breaking the cycle of immune suppression induced by tumor metabolism. Clinical trials combining metabolic therapy protocols with standard treatments are currently underway (NCT04730869) through analysis of metabolic characteristics within the glioma microenvironment. However, the precise efficacy remains unknown at present.

Additionally, dysregulated nucleotide metabolism is a hallmark of cancer. Beyond their fundamental roles in biosynthesis and proliferation, nucleotides are produced *via de novo* and salvage synthesis. The reprogramming of these pathways in tumors thereby contributes to aggressive proliferation, therapy resistance, and immune escape (Mullen and Singh, 2023). Altered nucleotide metabolism can suppress the anti-tumor activity of immune cells, such as T cells and macrophages, primarily through adenosine signaling (Mullen and Singh, 2023). Moreover, while the link between nucleotide metabolism and immunosenescence remains underexplored, targeting this metabolic axis represents a promising strategy for modulating the immune landscape in glioma. This potential, however, necessitates further investigation.

Concurrently, immunosenescence, an age-dependent decline of immune function, is profoundly correlated with various diseases, including tumors (Liu Z. et al., 2023). Researchers have documented the presence of senescent phenotypes across TAMs, T cells, NK cells, and B cells, characterized by functional exhaustion, telomere attrition, and a potent SASP (Goyani et al., 2024). These senescent immune cells are not merely inert; they become active architects of immunosuppression through the secretion of factors like IL-6, TGF-β, and IL-10. This creates a vicious cycle wherein the senescent TME further paralyzes effective anti-tumor immunity. Consequently, therapeutic strategies aimed at reversing immunosenescence—through the elimination of senescent cells (senolytics), neutralization of the SASP, or epigenetic rejuvenation (e.g., with DNMTis or HDACis)—represent a novel and complementary frontier in glioma therapy. Current research on immunosenescent features within the glioma microenvironment remains limited. Moreover, given the heterogeneous nature of gliomas, further studies are required to definitively characterize immunosenescence in this context. Elucidating the characteristics of immunosenescence within the glioma tumor microenvironment and combining these insights with standard-of-care treatments may offer a promising avenue to improve therapeutic outcomes.

Most critically, a bidirectional crosstalk exists between metabolic reprogramming and immunosenescence. Tumor-derived metabolic stress acts as a primary driver of immune cell senescence. In turn, the intrinsic metabolic alterations in senescent immune cells, such as impaired glycolysis and oxidative phosphorylation, further lock them into a dysfunctional state. This interplay forms a “vicious co-axis” that perpetuates immune evasion. However, as noted in the text, immunosenescence involves a decline in immune function and alterations in immune cell composition and activity, whose mechanisms overlap with those underlying immunosuppression (e.g., abnormal T cell function). In gliomas, a clear consensus is still lacking regarding the specific targets and temporal dynamics through which metabolic reprogramming regulates these two interconnected processes. Therefore, future efforts should focus on elucidating the direct mechanisms by which metabolic reprogramming governs immunosenescence in gliomas to establish a definitive link. This paradigm shift necessitates a move beyond monotherapies towards rational combination strategies. For instance, combining glycolytic inhibitors with interventions that reverse T cell senescence, or pairing immune checkpoint blockade with IDO1/TDO2 inhibition (Bickerdike et al., 2024), could yield synergistic efficacy by simultaneously targeting both the instigator (tumor metabolism) and the consequence (immune dysfunction) of this pathological network.

The BBB is a vital component of the CNS. It maintains a relatively independent internal environment within the CNS, distinct from other bodily systems, thereby ensuring normal physiological functions and a high degree of stability for neural activity. In gliomas, the physical and biochemical barriers of the BBB restrict the delivery of many therapeutic agents to the brain, contributing to suboptimal treatment outcomes for gliomas (Wang Y. H. et al., 2021). This limitation may also pose challenges for emerging therapeutic strategies targeting metabolic reprogramming and immunosenescence.

The future of glioma therapy lies in the development of intelligent combination strategies that integrate conventional radiochemotherapy, emerging immunotherapies, and novel metabolic and senolytic agents. Whether targeting metabolic reprogramming or immunosenescence, effective therapeutic strategies must fully account for the highly dynamic features of the glioma microenvironment. Targeting key metabolic nodes (e.g., GLUT, 2-HG), reversing immunosenescence, restoring immune function, and reshaping the immunosuppressive microenvironment all represent promising new directions. Furthermore, in-depth investigation into the interplay between metabolism and immunosenescence, and the subsequent design of combination therapies based on these insights, may open new avenues for glioma treatment.

In summary, this study elucidates the characteristics of metabolic reprogramming and immunosenescence in gliomas and reveals their potential interplay, thereby providing important insights for the future development of novel therapeutic strategies.
